# Hood’s Illustration of Hydropathy

**Published:** 1849-04

**Authors:** 


*Hood's Illustration of Hydropathy.*—"It has been our good fortune, since reading Claridge on Hydropathy, to see a sick drake avail himself of the 'water cure,' at the dispensary in Saint James Park. First, in wading in, he took a 'Fuse bad,' then took a 'Sitz bad,' and then turning his curly tail up in the air, he took a 'Kopf bad.' Lastly, he rose almost upright on his latter end, and made such a flapping with his wings, that we really expected he was going to shout 'Priesnitz for ever.' But no such thing. He only said, 'quack! quack!! quack!!!'"

